# A clinical-grade automated platform for the manufacturing of CAR-γδ T cells for immunotherapy

**DOI:** 10.3389/fimmu.2026.1779035

**Published:** 2026-05-22

**Authors:** Jan Kuska, Julia Kostyra, Lorraine Pinot, Evgeny Egorov, Nojan Jelveh, Lilian A. Martinez Carrera, Congcong Zhang, Svetlana Khorkova, Mario Assenmacher, Rimas Orentas, Sabine Mueller, José Villacorta Hidalgo

**Affiliations:** 1 Research and Development, Miltenyi Biotec B.V. & Co. KG, Bergisch Gladbach, Germany; 2 Department of Immunology, Institute for Cell Biology, University of Tübingen, Tübingen, Germany

**Keywords:** allogeneic cell therapy, cancer immunotherapy, gamma delta T cell transduction, gamma delta T cells, large scale manufacturing

## Abstract

Gamma delta (γδ) T cells are promising candidates for adoptive immunotherapy due to their unique biological and safety profiles. In particular, ex vivo expanded Vγ9Vδ2 T cells engineered with chimeric antigen receptors (CARs) have gained considerable interest, as they combine potent innate-like antitumor activity with a low risk of graft-versus-host disease and cytokine release syndrome, making them ideal for off-the-shelf cancer immunotherapies. However, as they represent only 1–5% of peripheral T cells, effective activation and expansion protocols are essential to generate sufficient numbers for clinical use. In this study, we describe an automated and highly efficient process for the expansion of Vγ9Vδ2 T cells and the manufacturing of CAR γδ T cells using the CliniMACS Prodigy closed system. This platform integrates cell separation, activation, genetic modification and expansion ensuring reproducibility and compliance with GMP standards. Starting with an alpha-beta (αβ) T cell and B cell magnetic depletion, we achieved a 4.59-log αβT cell depletion and a 3.97-log B cell depletion. By carefully optimizing activation, transduction and expansion, we were able to achieve up to 374-fold increase in Vγ9Vδ2 T cells, resulting in an average number of 6.64 x 10^9^ (4.76×10^9^ – 8.98×10^9^) cells after 14 days of culture using the TCT-LS process. Using a GMP-grade lentiviral vector pseudotyped with the envelope protein from baboon endogenous virus (BaEV) encoding a CD123 chimeric antigen receptor (CAR) at a multiplicity of infection (MOI) of 0.12, we achieved a mean γδ T cell transduction efficiency of 57.4%. Flow cytometry analysis of the final cell product showed an average composition of 89.83% γδ T cells, 9.82% NK cells, and 0.012% residual αβ T cells. The low average vector copy number (VCN) of 1.4 underscores the safety and efficiency of the transduction protocol. Functional assays against a leukemic cell line confirmed robust anti-tumor activity of CAR-γδ T cells produced using this platform, demonstrating the feasibility of large-scale manufacturing and highlighting their potential for future clinical immunotherapy applications.

## 1 Introduction

Adoptive cell therapies based on chimeric antigen receptor (CAR)-T cells and tumor-infiltrating lymphocytes (TILs) have transformed cancer treatment, particularly for blood cancers and some solid tumors (1, 2). However, a major challenge in treating tumors that lose or lack target antigens or HLA molecules is that adoptive cell therapies become ineffective as tumor cells evade immune detection, resulting in treatment failure and disease relapse (3, 4). In this context, γδ T cells have emerged as a promising alternative or complementary platform for adoptive cell therapy (5).

Unlike conventional αβ T cells, γδ T cells recognize cancer cells independently of MHC molecules, reducing the risk of graft-versus-host disease and enabling the use of allogeneic cell products. Their ability to detect stress-induced ligands on diverse tumor types, including those that evade αβ T cell recognition by downregulating HLA, lowers the chance of tumor immune escape (6). In human peripheral blood, the predominant γδ T cell subset is characterized by the expression of a T cell receptor (TCR) heterodimer comprising Vγ9 and Vδ2 variable chains (7). These lymphocytes specifically recognize the accumulation of phosphoantigens in cancer cells, such as isopentenyl pyrophosphate (IPP), through butyrophilin molecule-mediated recognition (8). IPP is produced by the mevalonate pathway, which is active in all higher eukaryotic cells. In contrast, some bacteria, such as *Mycobacterium tuberculosis*, and protozoan parasites, including those causing malaria, synthesize phosphoantigens via the non-mevalonate pathway (9). Interestingly, human Vγ9Vδ2 T cells are sensitive to very low concentrations of microbial phosphoantigens, allowing them to detect infected cells efficiently (10). In contrast, these T cells are much less responsive to phosphoantigens from the mammalian mevalonate pathway, preventing activation by normal cells. However, when mammalian cells are stressed or transformed, they upregulate phosphoantigen production to levels that can activate Vγ9Vδ2 T cells, highlighting their role in recognizing both infection and cellular stress (11).

The initial observation that aminobisphosphonates not only improve pain scores and quality of life in patients with bone metastases but also can stimulate Vγ9Vδ2 T cells provided a strong rationale to investigate their use beyond skeletal conditions (12). Widely prescribed for pathological bone disorders such as osteoporosis and cancer-associated metastases, these drugs have been shown to activate and expand Vγ9Vδ2 T cells, supporting their use for adoptive cell therapy in combination with cytokines such as IL-2 and IL-15 (13, 14). Aminobisphosphonates, such as pamidronate and zoledronate, inhibit the key enzyme farnesyl pyrophosphate synthase (FPPS) in the isoprenoid biosynthesis pathway. This inhibition leads to the intracellular accumulation of isopentenyl pyrophosphate (IPP), recognized by Vγ9Vδ2 T cells. When peripheral blood mononuclear cells (PBMCs) are cultured with zoledronate and cytokines, monocytes exhibit a pronounced accumulation of IPP, providing a potent activation signal for Vγ9Vδ2 T cells (15). Upon activation, Vγ9Vδ2 T cells undergo robust proliferation and exhibit enhanced cytotoxicity against a broad spectrum of tumor cells, accompanied by the production of pro-inflammatory cytokines such as IFN-γ and TNF-α (16, 17). Their natural cytotoxic capacity can be further augmented by engineering these cells to express chimeric antigen receptors (CARs) (18).

CAR-γδ T cells combine the innate tumor-targeting properties of Vγ9Vδ2 T cells with the antigen specificity conferred by CAR technology. This dual-targeting capability, together with their ability to recognize tumors in a HLA independent manner, overcomes key limitations associated with conventional αβ T cell therapies (19, 20).

We present here a GMP-compliant platform for the generation of CAR-Vγ9Vδ2 T cells using the CliniMACS Prodigy^®^system. This application demonstrates the feasibility of large-scale automated manufacturing of CAR-γδ T cells with a fully integrated and closed system, enabling scalable production for clinical applications. Our approach includes cell selection, activation, transduction, expansion and formulation, ensuring reproducibility and compliance with regulatory standards. Therefore, this platform represents a substantial advancement toward the clinical translation of γδ T cell therapies within the rapidly evolving landscape of cancer immunotherapy.

## 2 Materials and methods

### 2.1 Depletion of αβ T cells and B cells

Fresh leukapheresis products were obtained from adult healthy donors after written informed consent and in accordance with the World Medical Association (WMA) Declaration of Helsinki on ethical principles for medical research involving human subjects. Ethical approval was obtained from the Ethics Committee Uniklinik Ulm, Germany (approval number 172/99). As initial manufacturing step, αβ T cells and B cells were depleted using the CliniMACS Prodigy platform (Miltenyi Biotec). Leukapheresis products were incubated with the CliniMACS TCRα/β-Biotin reagent in combination with the Anti-Biotin Reagent to label αβ T cells. Concurrently, B cells were targeted using the CliniMACS CD19 Reagent (all reagents from Miltenyi Biotec). Cell separation was subsequently performed in a CliniMACS Tubing Set 320 (Miltenyi Biotec) using the LP-TCRab-19-45RA depletion process.

### 2.2 γδ T cell activation and expansion

A total of 1×10^9^ cells from the depleted material were seeded into a CliniMACS Tubing Set 620 and processed using the TCT-LS software. A customized culture matrix optimized for Vγ9Vδ2 T cells was employed to promote robust proliferation of these lymphocytes over a 14-day culture period. Cells were initially cultured in TexMACS GMP medium supplemented with 5% human AB serum, 500 IU/mL interleukin-2 (IL-2), 140 IU/mL interleukin-15 (IL-15), and 2 µg/mL zoledronate (Zometa^®^, Novartis). Following an initial pulse activation step, zoledronate was removed from the culture medium after 3 days by replacing the medium. The γδ T cells were subsequently maintained in TexMACS GMP medium with the same supplement of 5% human AB serum, 500 IU/mL IL-2, and 140 IU/mL IL-15 for the remainder of the 14-day culture period, with regular monitoring of cell viability, pH, lactate and glucose levels.

### 2.3 CAR-γδ T cell manufacturing

A second-generation, GMP-grade CD123 CAR containing a 4-1BB derived co-stimulatory domain and a CD3ζ signaling domain was evaluated (34). On day three of culture, the γδ T cells were transduced using baboon endogenous virus envelope protein pseudotyped-lentiviral particles (BaEV) diluted in serum-free medium and combined with MACS GMP Vectofusin-1 (Miltenyi Biotec). Producing BaEV requires this single change in envelope protein (ENV), all other components are identical to VSV-G pseudotyped lentivirus (LV) (35). After 7 minutes of incubation with Vectofusin-1, the vector particles were added to the cells with a multiplicity of infection (MOI) of 0.12. Spinoculation was performed for 2 h at 32 °C and 400×g to enhance the transduction efficiency. 24 hours after the transduction, the lentiviral particle-containing medium was removed.

### 2.4 Flow cytometry analysis

An in-process control (IPC) of the cell product was conducted every 2–4 days during the culture period to monitor the cell count and composition by using fluorochrome-conjugated antibodies anti-human CD45-VioBlue (REA747), anti-TCRγ/δ-APC (REA591), anti-CD14-VioGreen (REA599), anti-TCRα/β-PE (BW242/412), anti-CD3-FITC (REA613), anti-CD15-PerCP-Vio700 (VIMC6), anti-CD56-PE-Vio 670 (REA196) and anti-CD20-APC-Vio 770 (REA780) from Miltenyi Biotec. Additionally, a quality control (QC) of the final cell product was performed to analyze the γδ T cell phenotype using anti-TCR Vδ2-VioBlue (REA771), anti-CD8-VioGreen (REA734), anti-TCR Vδ1-FITC (REA173), anti-TCR Vγ9-APC-Vio 770 (REA470), anti-HLA-DR-FITC (REA805), anti-CD45RA-VioGreen (REA1047), anti-CD27-PE (REA499), anti-CD69-APC-Vio 770 (REA824), anti-KIR2DL1-VioGreen (REA1042), anti-TIM3-FITC (REA635), anti-PD1-PE (REA1165) and anti-TIGIT-PE-Vio 615 (REA1004), all from Miltenyi Biotec (Example of gating strategies used for flow cytometry analysis is presented in **Supplementary Figure 8**). To determine the CAR-transduction efficacy, the CD123 CAR detection reagent-Biotin was used in combination with the anti-Biotin-Vio^®^Bright B515 antibody (REA746) (Miltenyi Biotec). In all cases 7-AAD staining solution (Miltenyi Biotec) was used to assess cell viability and exclude dead cells. Flow cytometric analysis was carried out on a MACSQuant^®^ Analyzer 10 (Miltenyi Biotec) using MACSQuantify Software version 2.13. A comprehensive list of all reagents (**Table 1**), instruments (**Table 2**) and Flow cytometry panels (**Table 3**) is provided in the **Supplementary Material**.

### 2.5 *In vitro* cytotoxicity assessment

Transduced γδ T cells were co-cultured with the human lymphoblastic acute leukemia cell line RS4;11 (DSMZ Germany) in RPMI 1640 medium supplemented with 2 mM L-Glutamine and 10% fetal bovine serum (FBS). To assess the functional activity of CD123 CARγδ T cells, RS4;11 cells were genetically engineered to express the target antigen CD123 by lentiviral transduction. In addition, tumor cell lines were modified to express green fluorescent protein (GFP) to enable detection by flow cytometry. Co-cultures were incubated for 24 hours at effector-to-target (E:T) ratios from 5:1 to 0.3:1. Following incubation, target cell viability was determined by propidium iodide staining (Miltenyi Biotec). Cytotoxicity was quantified by measuring the GFP fluorescence intensity of viable target cells using the MACSQuant^®^ Analyzer 10 flow cytometer (Miltenyi Biotec) (Equation 1).


$$Specific\;lysis=100-\left(\frac{co\;culture\;tumor\;cell\;count\left(GFP^{+}PI^{-}cells\right)}{mono\;culture\;tumor\;cell\;count\left(GFP^{+}PI^{-}cells\right)}*100\right)$$

Equation 1: Assessment of the specific lysis of target cells within the *in vitro* cytotoxicity test.

### 2.6 Metabolic parameters

Medium parameters during large-scale culture were monitored by measuring glucose, lactate, and pH at regular intervals. Glucose concentrations were determined using a GlucCell glucose meter (Cesco Bioengineering). Lactate levels were measured with an Accutrend Plus system (Roche) in conjunction with BM-Lactate test strips (Roche). pH was assessed using a Seven2Go pH meter (Mettler-Toledo).

### 2.7 Vector copy number

To ensure the safety of the generated CAR-Vγ9Vδ2 T cells, the vector copy number (VCN) of the final cell product was determined. For this purpose, 1×10^6^ cells of the final cell product were stored at -20 °C as a cell pellet. Genomic DNA was isolated using the DNeasy Blood & Tissue Kit (Qiagen) following manufacturer’s instructions. After isolating genomic DNA from the transduced cells, and VCN was subsequently quantified with the MACS^®^ COPYcheck Kit (Miltenyi Biotec), also following the manufacturer’s protocol.

### 2.8 RNA extraction and bulk γδ TCR sequencing

To investigate the effect of the expansion process on the TCR repertoire, we performed bulk-RNA sequencing on cells derived from the αβ T cell- and B cell-depleted starting population as well as from the final cell product. This approach allowed us to track the dynamics of Vγ9Vδ2 T cells responding to zoledronic acid stimulation. RNA was extracted using RNeasy Mini Kit (QIAGEN), RNA yield was quantified using Qubit RNA HS Assay kit (Thermo Fischer Scientific) and quality was assessed with RNA 6000 Nano Chip (Agilent). For the bulk gamma and delta TCR sequencing, libraries were generated using the MiLab Human g & d TCR RNA Multiplex Kit (MiLaboratories). The libraries were quantified by Qubit dsDNA HS Assay kit (Thermo Fischer Scientific), Bioanalyzer High sensitivity DNA Assay (Agilent), and quantitative PCR with the NEB Next Library Quant Kit for Illumina (New England Biolabs). Sequencing was performed on Illumina MiSeq as QC run, and finally on Illumina NextSeq 2000.

### 2.9 Bioinformatics analysis

MiXCR v. 4.6.0. with recommended preset “milab-human-rna-tcr-umi-multiplex” was used to address quality control and assemble TCR clonotypes from raw fastq files (21). TCR clonotypes were defined based on unique nucleotide CDR3 sequences and subsequently filtered to retain only productive clones. Downstream analyses were carried out in R v4.1.3 using the tidyverse v1.3.2. To compare the UMI proportions of the top 20 clonotypes in cultivated T cell samples against their original frequencies in PBMC samples, we used Immunarch v0.9.1. Clonotypes significantly enriched during cultivation were identified using edgeR v3.40.1, applying thresholds of FDR< 0.05 and log fold change > 2. Volcano plots, gene usage bar plots and histograms of CDR3 amino acid length were generated with ggplot2 v. 3.5.1. The relationship between cultivation and gene usage was assessed using Pearson’s Chi-squared test. To evaluate the similarity of CDR3 amino acid sequences of enriched clonotypes we used igraph v. 2.1.1, connecting clonotypes that differed by one or fewer amino acid mismatches. We further filtered TRG clonotypes that had at least one neighbor in the network and possessed the most frequent lengths (16 or 17 amino acids) for more detailed analysis. These sequences were aligned using msa v1.26.0, and seqLogo v1.60.0 was used to generate sequence logos representing amino acid frequency patterns.2.9 Statistical analysis.

### 2.10 Statistical analysis and data availability

All statistical analysis were performed using GraphPad PRISM version 10 (GraphPad Software, Inc., San Diego, CA, USA). Results are shown as the mean ± standard deviation of the mean.

Sequencing data (FASTQ files) reported in this manuscript are available to download from https://zenodo.org/records/19359566.

## 3 Results

### 3.1 αβ T cell and B cell depletion performance

**Figure 1:**
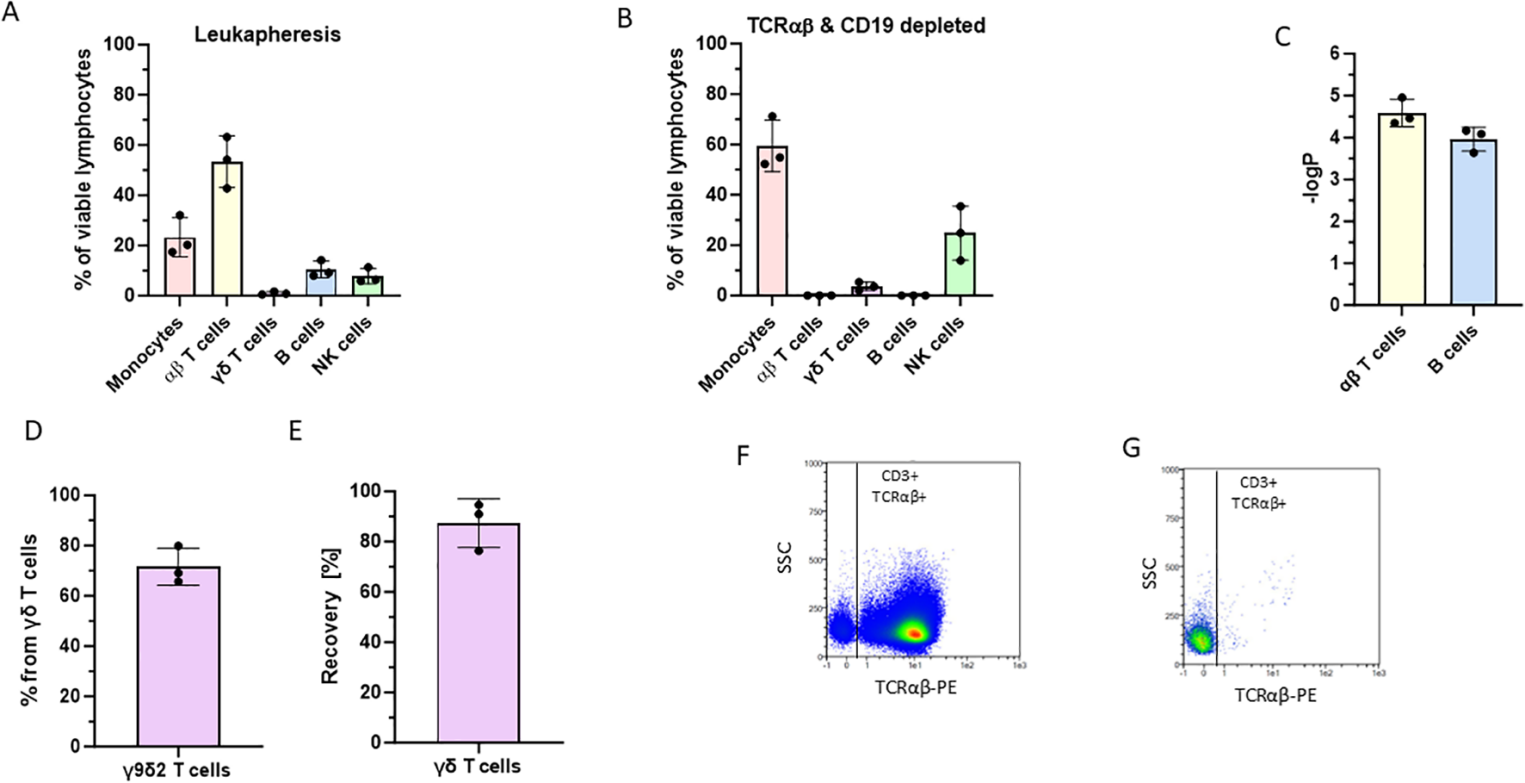
Depletion performance of αβ T and B cells using the LP-TCRab-19-45RA process on the CliniMACS Prodigy from three healthy donors. **(A)** Lymphocyte initial composition of the leukapheresis blood source. The percentages of different cell types were determined by flow cytometry. **(B)** Lymphocyte composition of the αβTCR/CD19 depleted cell product, composed mostly of monocytes and NK cells with a small fraction of γδ T cells. **(C)** Log depletion of the αβ T and B cells using the LP-TCRab-19-45RA that was integrated into our process. **(D, E)** Proportion of Vγ9Vδ2 T cells from the γδ T cell subset after the LP-TCRab-19-45RA process. Representative flow cytometry dot plots demonstrating the αβT cell composition pre **(F)** and post **(G)** depletion.

Fresh leukapheresis products from healthy donors were used for the depletion process. On average 52.91% αβ T cells and 10.18% B cells were initially present in the cell source (**Figure 1A**). After depletion, only 0.0068% αβ T cells and 0.0055% B cells remained, corresponding to a logarithmic depletion greater than 3.9 for B cells and 4.5 for αβT cells (**Figures 1B, C, F, G**). The depleted material was subsequently used for the expansion of γδ T cells during a 14-day culture period. On average, 3.67% of the seeded leukocytes were γδ T cells, and of this small population, 71.57% were Vγ9Vδ2 T cells that can be activated and expanded by zoledronate (**Figures 1D, E**).

### 3.2 Culture parameters for a large scale γδ T cell expansion

**Figure 2:**
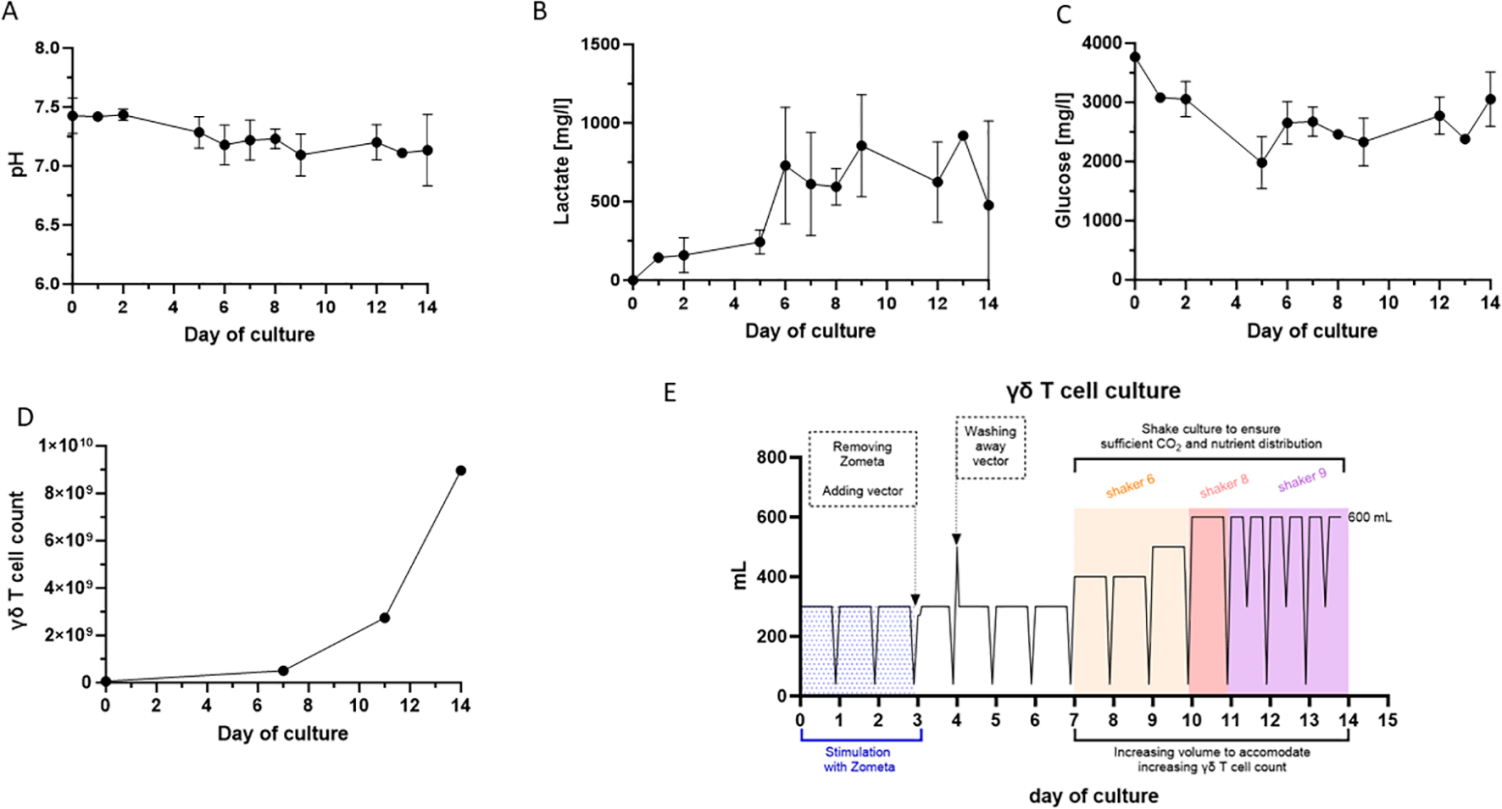
Culture parameters of the γδ T cell manufacturing protocol using the TCT-LS process on the CliniMACS Prodigy of three healthy donors. **(A)** Medium pH levels during the 14-day culture period. **(B)** Lactate levels in the culture medium during the 14-day culture period [mg/l]. **(C)** Glucose levels in the culture medium during the 14-day culture period [mg/l]. **(D)** Cell counts for γδ T cells over the 14-day culture period for one representative donor. **(E)** Automated γδ T cell feeding matrix using the CliniMACS TCT-LS software on the CliniMACS Prodigy. During the first week of culture, the medium is carefully replaced to promote cell-to-cell contact. In the second phase of the process, the culture volume was gradually increased to a final volume of 600 ml providing fresh medium for the exponential expansion. Gentle shaking of the culture chamber ensured homogeneous cell suspension and promoted the achievement of high cell densities. From day 10 onward, the medium was exchanged twice daily to sustain rapid proliferation. By day 14, cell densities of up to 1.5 × 10^7^ cells/ml were reached.

To enable stable and efficient expansion of Vγ9Vδ2 T cells, an automated feeding schedule was developed using the CliniMACS Prodigy T cell Transduction- Large scale (TCT LS). Due to the lymphocyte composition of the starting material, many bystander cells (e.g. monocytes) are present in the first days of the culture. Therefore, a sufficient medium exchange must be ensured. Preliminary experiments showed that the expansion and function of Vγ9Vδ2 T cells is impaired if the pH falls below 6.8 during culture. In addition, the glucose level should not fall below 2000 mg/l, and the lactate level should not exceed 1500 mg/l. This was verified by deferring medium exchanges until the predefined metabolite acceptance criteria had been met. Upon attainment of these values, the medium was replaced, and subsequent cell expansion and viability were systematically assessed. With a standardized culture feeding matrix, we were able to ensure that these critical parameters were maintained to promote the expansion of Vγ9Vδ2 T cells in the Prodigy system chamber (**Figures 2A–C**). Based on a starting cell density of 3.3×10^6^ leukocytes/ml, daily replacement of the medium is required to ensure stable expansion of the Vγ9Vδ2 T cells and removal of cell debris and metabolites from the culture. During the first week of culture, the medium is replaced without shaking to maximize cell-to cell contact, enabling Vγ9Vδ2 T cells to recognize the activation signals generated by zoledronate activity. As the γδ T cells start to expand exponentially in the second week of culture (**Figure 2D**), the culture is gradually replenished and increased to a final culture volume of 600 ml. Shaking of the culture chamber is used to keep cells in suspension, thus enabling very high cell densities. During peak expansion from day 10 onwards, the medium needs to be replaced twice per day to support the Vγ9Vδ2 T cells (a detailed diagram is provided in **Figure 2E** and the activity matrix in **Supplementary Table 4**). On day 14, a cell density of up to 1.5×10^7^ cells/ml was achieved.

### 3.3 γδ T cell culture and expansion

**Figure 3:**
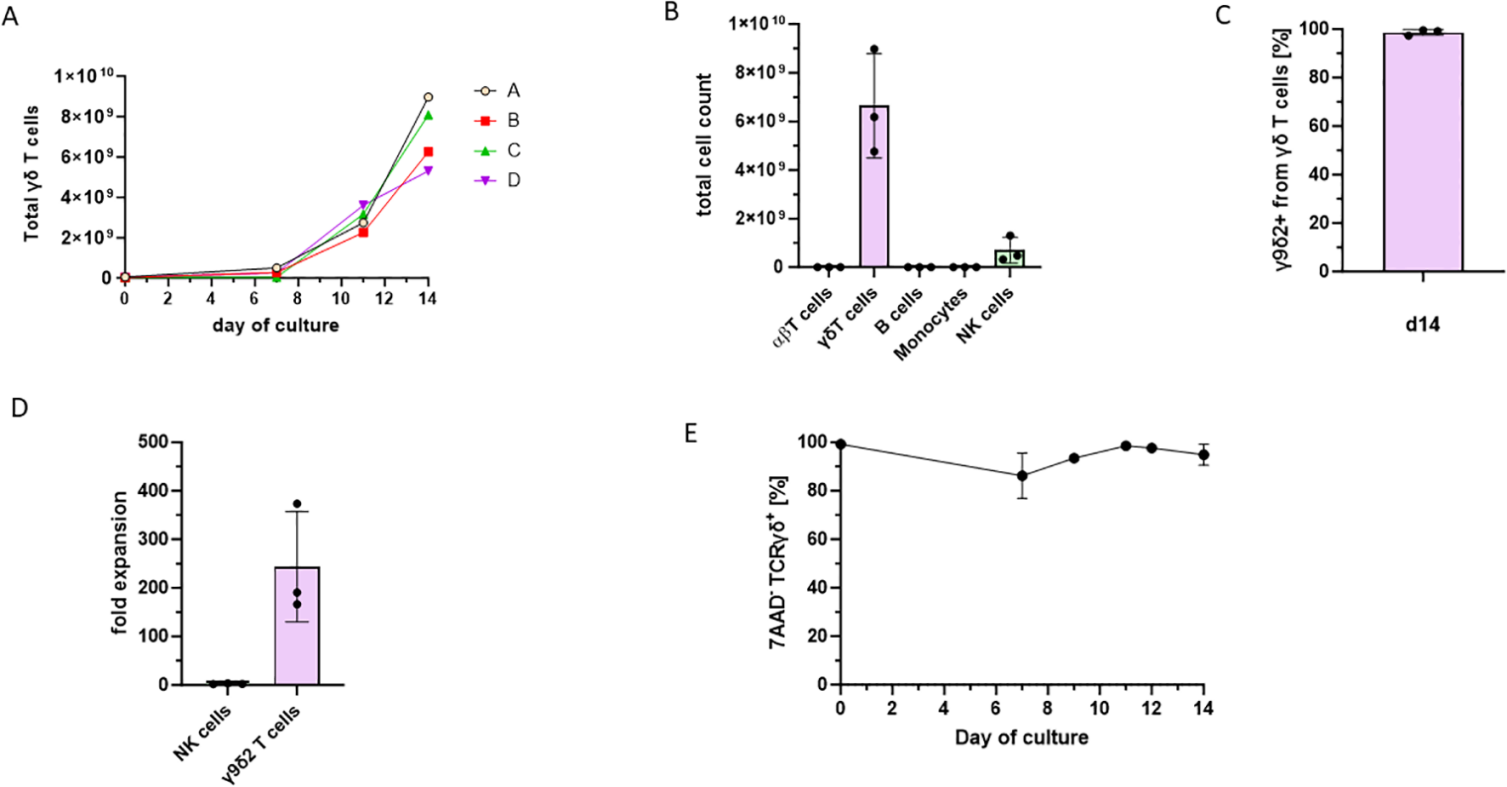
Final cell product of the γδ T cell manufacturing protocol using the TCT-LS process on the CliniMACS prodigy from four healthy donors. The cells were cultured for 14 days in the Prodigy system. The percentages of different cell types were determined by flow cytometry. **(A)** Total cell numbers after expansion of γδ T cells from four different expansion experiments with a marked expansion after day 6 of the process. Cell count was determined by flow cytometry. **(B)** Lymphocyte composition of the final cell product. **(C)** Proportion of Vγ9Vδ2 T cells from the γδ T cell subset of the final cell product. **(D)** Fold expansion of NK cells and Vγ9Vδ2 T cells after the 14-day culture period. **(E)** Viability of γδ T cells through the 14-day culture period.

Starting from a mean of 2.39×10^7^ Vγ9Vδ2 T cells per 1×10^9^ leukocytes, the γδ T cells exhibited rapid proliferation and became the predominant cell population within the first week of culture. On average, a 243.52-fold expansion of Vγ9Vδ2 T cells was achieved (**Figure 3A**). By the end of the culture period, the cell product consisted almost exclusively of γδ T cells, with a small fraction of remaining NK cells (**Figure 3B**), confirming Vγ9Vδ2 T cells as a dominant subset (**Figure 3C**). NK cells expanded 2.60-fold on average, resulting in a γδ T cell to NK cell ratio of 9.6:1, corresponding to a mean of 6.71×10^9^ γδ T cells and 7.01×10^8^ NK cells in the final cell product (**Figure 3D**). Throughout the culture period, γδ T cell viability remained stable and was consistent between donors (**Figure 3E**).

### 3.4 γδ T cellular product characterization

**Figure 4:**
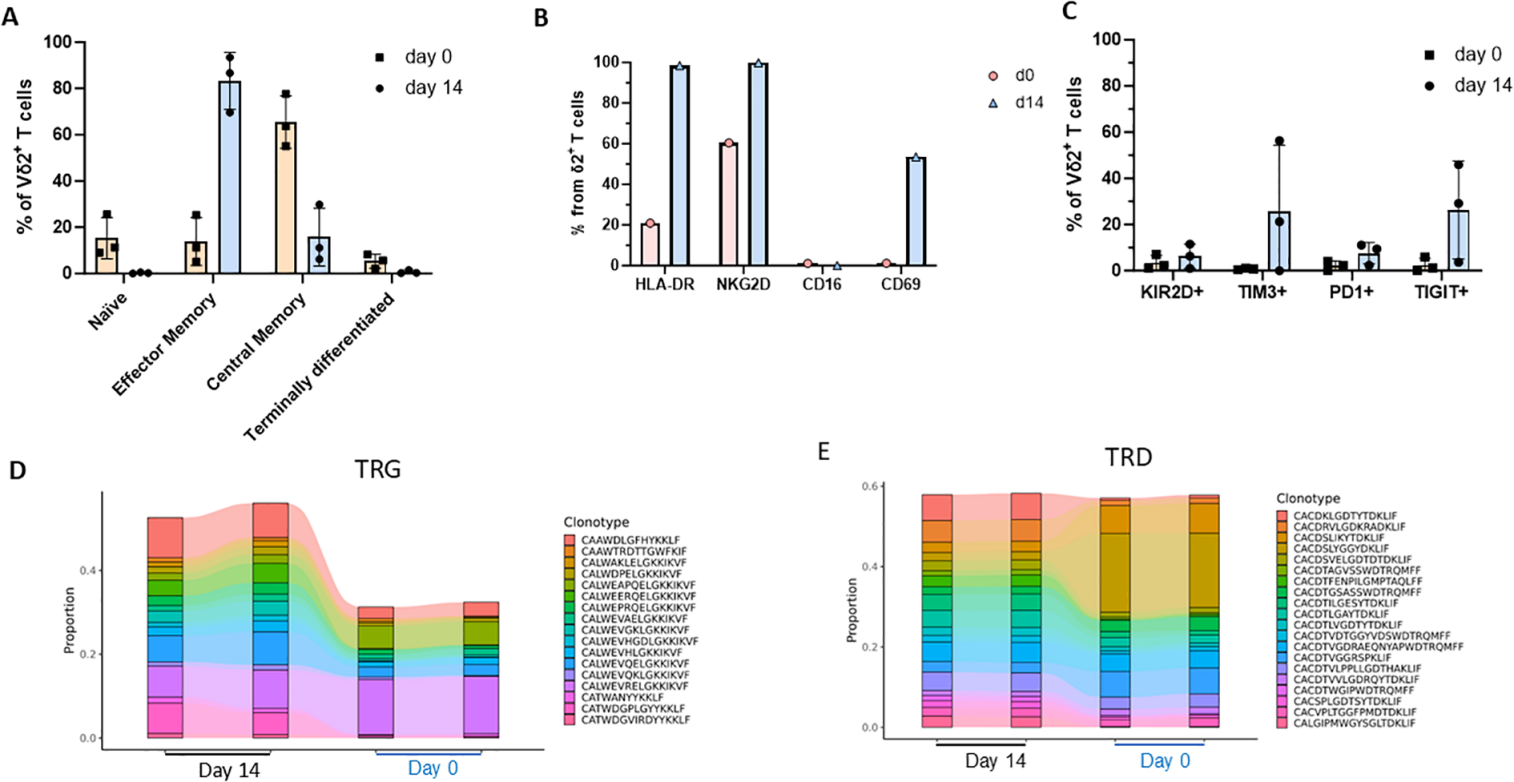
Characterization of the γδ T cells of three healthy donors. The cells were maintained in culture for 14 days in the Prodigy system with the γδ T cell manufacturing protocol using the TCT-LS process. The percentages of different marker expressions were determined by flow cytometry. **(A)** CD27 and CD45RA T-cell memory marker expression by Vδ2 T cells. The cells were assigned as naïve (CD27+CD45RA+), effector memory (CD27-CD45RA-), central memory (CD27+CD45RA-) and terminally differentiated (CD27-CD45RA+). **(B)** Activation markers expressed on Vγ9Vδ2 T cells from a representative expansion run showing a higher expression of NKG2D and HLA-DR with a lower expression of CD69 and a very low CD16 at Day 14 of culture. **(C)** Expression of different inhibitory receptors (KIR2D, TIM-3, PD-1, TIGIT) on Vδ2 T cells after 14 days of expansion. **(D)** Frequency distribution of the top 20 TRG and TRD clonotypes at day 0 (D0) and day 14 (D14) of culture. All top 20 clonotypes identified at D0 remained detectable and exhibited a more balanced frequency distribution at D14, indicating preservation of a polyclonal repertoire. Sequencing was performed in duplicate; each column represents clonotype frequency at D0 and D14 for TRG and TRD.

The phenotype and activation profile of γδ T cells effectively define their functional efficacy. At day 0 of culture, Vγ9Vδ2 T cells predominantly displayed a central memory and naïve phenotype, which progressively shifted toward a mixed central and effector memory phenotype by day 14 (**Figure 4A**). Despite inter-donor variability, a consistent predominance of effector memory cells was observed, with a mean frequency of 83.28%. Expression of characteristic Vγ9Vδ2 T-cell markers, including NKG2D and HLA-DR, was maintained in the final product, together with expression of the activation marker CD69 (**Figure 4B**). By day 14, inhibitory receptors were upregulated, with TIM-3 and TIGIT showing the most pronounced increase, whereas PD-1 expression remained comparatively lower (**Figure 4C**). High-throughput sequencing confirmed the polyclonal nature of the final γδ T-cell product. Comparative analysis of TCR clonotype repertoires between day 0 and day 14 demonstrated a dynamic restructuring, consistent with the selective expansion of clonotypes responsive to the culture conditions (**Figure 4D**). While most TCR clonotypes maintained stable frequencies throughout the cultivation period, a subset comprising 144 TRG and 58 TRD clonotypes became significantly enriched by day 14 (**Supplementary Figure 1**). Enriched TCRγ clonotypes predominantly utilized TRGV9* and TRGJP* gene segments, whereas enriched TCRδ clonotypes were mainly characterized by TRDV2* and TRDJ1* usage (**Supplementary Figure 2**). Compared with the overall repertoire, these TCRγ clonotypes displayed longer average CDR3 regions, suggesting potential selection for specific structural features during culture (**Supplementary Figure 3**). Analysis of CDR3 amino acid sequences from enriched clonotypes clustering those differing by ≤1 amino acid revealed a distinct cluster of highly similar TRG clonotypes; this pattern was absent in the TRD repertoire (**Supplementary Figures 4**, **5**). Consistent with these findings, the inverse Simpson index demonstrated higher and more evenly distributed TCRγ diversity at day 14 compared to day 0, reflecting balanced clonal expansion over time (**Supplementary Figure 6**).

### 3.5 Transduction of γδ T cells with a CD123 CAR

**Figure 5:**
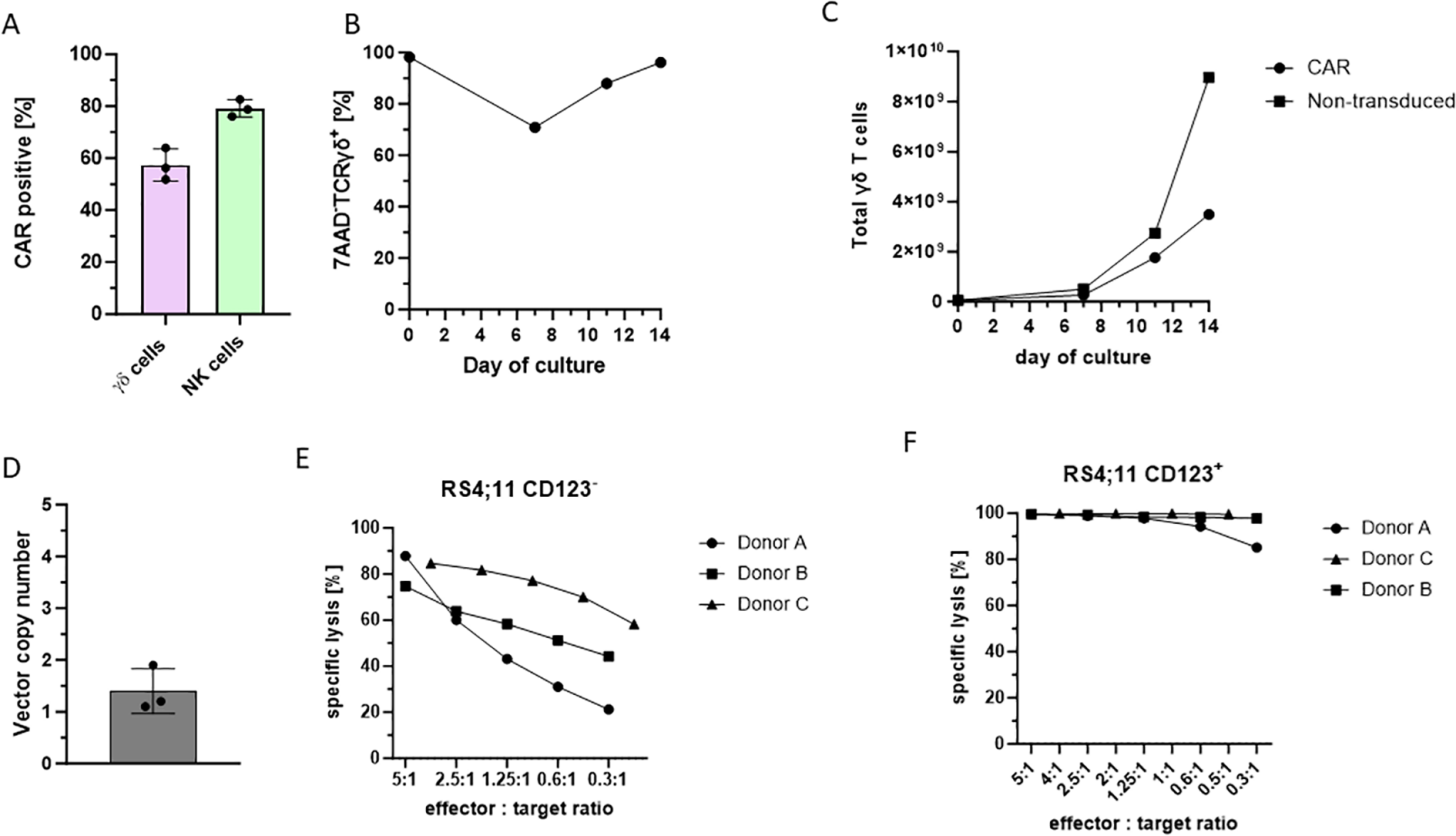
CAR modified cell product of the γδ T cell manufacturing protocol using the TCT-LS process on the CliniMACS Prodigy of three healthy donors. The cells were cultivated for 14 days in the Prodigy system. Transduction was performed on day 3 with the CD123-CAR BaEV (Miltenyi Biotec). The percentages of different cell types and transduction efficiency were determined by flow cytometry. **(A)** Transduction efficiency of γδ T cells and NK cells on day 14. **(B)** Viability of γδ T cells through the 14-day culture period of one representative donor. **(C)** Comparative cell counts for γδ T cells over the 14-day culture period of γδ T cells without transduction and CAR transduced cells. **(D)** Vector copy number of the CAR-modified cell product. **(E)** Specific lysis of RS4;11 CD123- cells. 24-hour co-culture with the CAR-modified cell product at different effector:target ratios of one donor. **(F)** Specific lysis of RS4;11 CD123+ cells. 24-hour co-culture with the CAR-modified cell product at different effector:target ratios of one donor.

To enhance the target cell recognition of the Vγ9Vδ2 T cells, an optimized transduction protocol for the CliniMACS Prodigy system was established. Given that the transduction procedure could impair the cell expansion, we titrated the lentiviral particles to find the best ratio between transduction efficiency and γδ T cell expansion (**Supplementary Figure 7**). Based on the described initial cell count and conditions, a MOI of 0.12 was optimal. In addition, Vectofusin-1 was used to enhance the transduction efficiency. These optimizations allowed us to obtain a mean transduction efficiency of 57.4% for the γδ T cells, and a 79.2% transduction efficiency for the lower number of NK cells remaining in the culture (**Figure 5A**). BaEV LV transduction on day 3 lowered the measured viability of the cultured γδ T cells in a transitory manner (**Figure 5B**). The cells expanding during the second culture week, evidenced a mean viability of 96.4% on day 14. Over the entire culture period, the transduced γδ T cells showed a consistent growth profile, using non-transduced cells as a comparator (**Figure 5C**). On average, 1.5×10^9^ γδ T cells (8.22×10^8^ CAR-γδ T cells) were obtained in the final cell product on day 14. The overall low VCN (vector copies per target cell genome) of 1.4 (1.1-1.9) underlines the safety of the generated CAR-γδ T cell and NK cell product (**Figure 5D**). In contrast to most NK cell transduction protocols, the incorporation of a spinoculation step did not enhance the transduction efficiency of γδ T cells (data not shown). Considering the high transduction efficiency achieved with the GMP-grade lentiviral vector in both NK and γδ T cells, the spinoculation step may be omitted to minimize cellular stress, particularly during the initial culture phase.

To investigate the anti-tumor efficacy of the CAR-modified cell product, an *in vitro* killing assay was performed. The RS4;11 cell line, genetically modified to express the target antigen CD123, was co-cultured with the CAR-γδ T cell product for 24 hours. As a control, unmodified RS4;11 cells were used to determine the non-CAR mediated cytotoxicity of the cell product. The cell product showed a dose dependent anti-tumor cytotoxicity against the RS4;11 CD123 negative cells (**Figure 5E**). The killing capacity was enhanced against CD123+ target cells, leading to a specific lysis of >85% even with an E:T ratio as low as 0.3:1 (**Figure 5F**).

## 4 Discussion

To enable the clinical translation of Vγ9Vδ2 T cells, robust and scalable manufacturing under GMP-compliant conditions is essential. During the 14-day cell manufacturing campaign we developed, Vγ9Vδ2 T cells underwent a phenotypic transition from naïve and central memory subsets toward an effector-differentiated state, reflecting progressive functional maturation and acquisition of cytotoxic competence (22, 23). These findings align with previous studies demonstrating the potent cytotoxic activity of γδ T cells and their ability to target a broad range of tumor cells through recognition of non-peptide antigens expressed by malignant cells (24). Selective depletion of alloreactive αβ T cells and B cells has been reported to minimize acute and chronic GvHD, outperforming T cell-replete grafts and enabling haploidentical or mismatched HSCT without extensive immunosuppression (25). Conversely, the retention of γδ T cells and NK cells supports rapid engraftment, improved relapse-free survival, reduced non-relapse mortality, and lower infection rates (26, 27). These findings emphasize the dual role of γδ T cells in promoting immune reconstitution while mitigating GvHD risk.

Given the unique characteristics of γδ T cells and NK cells, different groups have developed protocols to expand γδ T cells using various stimulation methods and culture platforms for the clinical use of Vγ9Vδ2 T cells, often in combination with chemotherapeutic agents or monoclonal antibodies (28–31). In this study, we demonstrate the feasibility of using the CliniMACS Prodigy closed system for the automated, large-scale generation of highly functional Vγ9Vδ2 T cells. This approach enables a harmonized and scalable manufacturing process, facilitating broader clinical implementation and extending the use beyond transplantation settings.

Our group has previously shown successful lentiviral transduction of γδ T cells using the baboon envelope pseudotyped system (BaEV) to generate CAR-modified γδ T cells with strong anti-tumor reactivity (35). In this study, CAR-transduced γδ T cells specifically targeted and killed CD123^+^ tumor cells with high transduction efficiency and viability. The low vector copy number (VCN) of the engineered cells highlights the safety and quality of the generated CAR γδ T cell product. This strategy effectively combines the innate-like tumor recognition of γδ T cells with CAR-mediated antigen-specific recognition, enhancing their therapeutic potential. This model could facilitate point-of-care manufacturing, reduce production costs, and increase accessibility of advanced cell therapies (32).

Despite these promising outcomes, donor-to-donor variability remains a critical challenge, which could affect expansion rates and functional performance. Factors such as donor age, health status, and genetic background may contribute to these differences (33). Standardized manufacturing protocols and optimization of culture conditions will be essential to minimize variability and ensure consistent therapeutic quality. Future research should focus on addressing donor variability, integrating immune checkpoint modulation, and exploring rational combination therapies to maximize the therapeutic efficacy of γδ T cells.

In conclusion, our study establishes a robust, automated process for large-scale Vγ9Vδ2 T cell production using the CliniMACS Prodigy platform. By integrating steps from cell selection through final formulation, this system enables the generation of a high-quality allogeneic cell product suitable for cancer immunotherapy. The capacity for CAR modification and the potential reduction in GVHD risk further underscore the translational promise of γδ T cell–based therapies.

## Data Availability

The original contributions presented in the study are included in the article/**Supplementary Material**, further inquiries can be directed to the corresponding author/s.

## Supplementary material

Supplementary Figure 1 The majority of TCR clonotypes maintained stable frequencies throughout cultivation. However, a subset showed significant enrichment by day 14, including 144 TRG and 58 TRD clonotypes.

Supplementary Figure 2 Analysis of the enriched TCR gamma clonotypes predominantly utilized TRGV9 and TRGJP gene segments, while the enriched TCR delta clonotypes were mainly characterized by TRDV2 and TRDJ1 usage.

Supplementary Figure 3 Enriched TCR gamma clonotypes exhibited a longer average CDR3 length compared to the complete repertoire, suggesting potential selection for specific structural features during cell culture.

Supplementary Figure 4 A similarity graph based on the CDR3 amino acid sequences of enriched clonotypes was constructed, where clones differing by one or fewer amino acid mismatches were connected. Within the TRG repertoire, a distinct cluster of highly similar clonotypes was observed, indicating potentially shared antigen specificity.

Supplementary Figure 5 The CDR3 amino acid frequency was analyzed for enriched clonotypes that had at least one neighbor in the similarity graph, highlighting conserved sequence features among closely related clones.

Supplementary Figure 6 The inverse simpson index revealed that the diversity of TCR gamma clonotypes was higher and more evenly distributed on day 14 of cultivation compared to day 0, indicating balanced clonal expansion over time.

Supplementary Figure 7 CAR transduction efficiency on Vγ9Vδ2 T cells at different multiplicity of infection (MOI) was tested to define the process.

Supplementary Figure 8 Gating strategy used for the flow cytometry analysis of leukapheresis cell composition **(A)**, cell composition after depletion **(B)**, and phenotype of the depleted product **(C)**.

## References

[1] Patel K Tariveranmoshabad M Kadu S Shobaki N June C . From concept to cure: The evolution of CAR-T cell therapy. Mol Ther. (2025) 33:2123–40. doi: 10.1016/j.ymthe.2025.03.005. PMID: 40070120 PMC12126787

[2] Matsueda S Chen L Li H Yao H Yu F . Recent clinical research and technological development in TIL therapy. Cancer Immunol Immunother. (2024) 73:232. doi: 10.1007/s00262-024-03793-4. PMID: 39264449 PMC11393248

[3] Sterner RC Sterner RM . CAR-T cell therapy: current limitations and potential strategies. Blood Cancer J. (2021) 11:69. doi: 10.1038/s41408-021-00459-7. PMID: 33824268 PMC8024391

[4] Khong HT Wang QJ Rosenberg SA . Identification of multiple antigens recognized by tumor-infiltrating lymphocytes from a single patient: tumor escape by antigen loss and loss of MHC expression. J Immunother. (2004) 27:184–90. doi: 10.1097/00002371-200405000-00002. PMID: 15076135 PMC2275330

[5] Xu Y Xiang Z Alnaggar M Kouakanou L Li J He J . Allogeneic Vγ9Vδ2 T-cell immunotherapy exhibits promising clinical safety and prolongs the survival of patients with late-stage lung or liver cancer. Cell Mol Immunol. (2021) 18(2):427–39. doi: 10.1038/s41423-020-0515-7. PMID: 32939032 PMC8027668

[6] Chien YH Jores R Crowley MP . Recognition by gamma/delta T cells. Annu Rev Immunol. (1996) 14:511–32. doi: 10.1146/annurev.immunol.14.1.511. PMID: 8717523

[7] Davodeau F Peyrat MA Hallet MM Gaschet J Houde I Vivien R . Close correlation between Daudi and mycobacterial antigen recognition by human gamma delta T cells and expression of V9JPC1 gamma/V2DJC delta-encoded T cell receptors. J Immunol. (1993) 151:1214–23. doi: 10.4049/jimmunol.151.3.1214. PMID: 8393042

[8] Rigau M Ostrouska S Fulford TS Johnson DN Woods K Ruan Z . Butyrophilin 2A1 is essential for phosphoantigen reactivity by γδ T cells. Science. (2020) 367:eaay5516. doi: 10.1126/science.aay5516. PMID: 31919129

[9] Constant P Davodeau F Peyrat MA Poquet Y Puzo G Bonneville M . Stimulation of human gamma delta T cells by nonpeptidic mycobacterial ligands. Science. (1994) 264:267–70. doi: 10.1126/science.8146660. PMID: 8146660

[10] Tanaka Y Morita C Tanaka Y Nieves E Brenner MB Bloom BR . Natural and synthetic non-peptide antigens recognized by human γδ T cells. Nature. (1995) 375:155–8. doi: 10.1038/375155a0. PMID: 7753173

[11] Thedrez A Sabourin C Gertner J Devilder MC Allain-Maillet S Fournié JJ . Self/non-self discrimination by human gammadelta T cells: simple solutions for a complex issue? Immunol Rev. (2007) 215:123–35. doi: 10.1111/j.1600-065X.2006.00468.x. PMID: 17291284

[12] Kunzmann V Bauer E Wilhelm M . Gamma/delta T-cell stimulation by pamidronate. N Engl J Med. (1999) 340:737–8. doi: 10.1056/NEJM199903043400914. PMID: 10068336

[13] Wardley A Davidson N Barrett-Lee P Hong A Mansi J Dodwell D . Zoledronic acid significantly improves pain scores and quality of life in breast cancer patients with bone metastases: a randomised, crossover study of community vs hospital bisphosphonate administration. Br J Cancer. (2005) 92:1869–76. doi: 10.1038/sj.bjc.6602551. PMID: 15870721 PMC2361764

[14] Van Acker HH Anguille S Willemen Y Van den Bergh JM Berneman ZN Lion E . Interleukin-15 enhances the proliferation, stimulatory phenotype, and antitumor effector functions of human gamma delta T cells. J Hematol Oncol. (2016) 9:101. doi: 10.1186/s13045-016-0329-3. PMID: 27686372 PMC5041439

[15] Kondo M Sakuta K Noguchi A Ariyoshi N Sato K Sato S . Zoledronate facilitates large-scale ex vivo expansion of functional gammadelta T cells from cancer patients for use in adoptive immunotherapy. Cytotherapy. (2008) 10:842–56. doi: 10.1080/14653240802419328. PMID: 19016372

[16] Saura-Esteller J de Jong M King LA Ensing E Winograd B de Gruijl TD . Gamma delta T-cell based cancer immunotherapy: past-present-future. Front Immunol. (2022) 13:915837. doi: 10.3389/fimmu.2022.915837. PMID: 35784326 PMC9245381

[17] Subhi-Issa N Tovar Manzano D Pereiro Rodriguez A Sanchez Ramon S Perez Segura P Ocaña A . γδ T cells: game changers in immune cell therapy for cancer. Cancers. (2025) 17:1063. doi: 10.3390/cancers17071063. PMID: 40227601 PMC11987767

[18] Capsomidis A Benthall G Van Acker HH Fisher F Kramer AM Abeln Z . Chimeric antigen receptor-engineered human gamma delta T cells: enhanced cytotoxicity with retention of cross presentation. Cell. (2018) 26:354–65. doi: 10.1016/j.ymthe.2017.12.001. PMID: 29310916 PMC5835118

[19] Hu Y Hu Q Li Y Lu L Xiang Z Yin Z . γδ T cells: origin and fate, subsets, diseases and immunotherapy. Sig Transduct Target Ther. (2023) 8:434. doi: 10.1038/s41392-023-01653-8. PMID: 37989744 PMC10663641

[20] Subhi-Issa N Tovar Manzano D Pereiro Rodriguez A Sanchez Ramon S Perez Segura P Ocaña A . γδ T cells: game changers in immune cell therapy for cancer. Cancers. (2025) 17:1063. doi: 10.3390/cancers17071063. PMID: 40227601 PMC11987767

[21] Bolotin DA Poslavsky S Mitrophanov I Shugay M Mamedov IZ Putintseva E . MiXCR: software for comprehensive adaptive immunity profiling. Nat Methods. (2015) 12:380–1. doi: 10.1038/nmeth.3364. PMID: 25924071

[22] Dieli F Poccia F Lipp M Sireci G Caccamo N di Sano C . Differentiation of effector/memory Vdelta2 T cells and migratory routes in lymph nodes or inflammatory sites. J Exp Med. (2003) 198:391–7. doi: 10.1084/JEM.20030235. PMID: 12900516 PMC2194087

[23] Giannotta C Autino F Massaia M . Vg9Vd2 T-cell immunotherapy in blood cancers: ready for prime time? Front Immunol. (2023) 14:1167443. doi: 10.3389/fimmu.2023.1167443. PMID: 37143664 PMC10153673

[24] Gomes AQ Correia DV Grosso AR Lança T Ferreira C Lacerda JF . Identification of a panel of ten cell surface protein antigens associated with immunotargeting of leukemias and lymphomas by peripheral blood γδ T cells. Haematologica. (2010) 95:1397–404. doi: 10.3324/haematol.2009.020602. PMID: 20220060 PMC2913090

[25] Bethge WA Eyrich M Mielke S Meisel R Niederwieser D Schlegel PG . Results of a multicenter phase I/II trial of TCRαβ and CD19-depleted haploidentical hematopoietic stem cell transplantation for adult and pediatric patients. Bone Marrow Transplant. (2022) 57:423–30. doi: 10.1038/s41409-021-01551-z. PMID: 34952929 PMC8702395

[26] Abdelgawad HAH Aboeldahab H Belal MM Bashir MN Miller HK Handgretinger R . Comprehensive up-to-date analysis on TCRαβ/CD19-depleted hematopoietic stem cell transplantation in pediatric hematological Malignancies. Transpl Immunol. (2025) 90:102220. doi: 10.1016/j.trim.2025.102220. PMID: 40107625

[27] Stuut AHG Nijssen C van der Wagen L van Rhenen A Daenen LGM Janssen A . Improved GVHD-free and relapse-free survival after ex vivo αβTCR and CD19 depleted allogeneic HSCT compared to T cell replete HSCT. Bone Marrow Transplant. (2025) 60(5):673–81. doi: 10.1038/s41409-025-02538-w. PMID: 40089614

[28] Peters C Kouakanou L Oberg HH Wesch D Kabelitz D . *In vitro* expansion of Vγ9Vδ2 T cells for immunotherapy. Methods Enzymol. (2020) 631:223–37. doi: 10.1016/bs.mie.2019.07.019. PMID: 31948549

[29] Bold A Gross H Holzmann E Knop S Hoeres T Wilhelm M . An optimized cultivation method for future *in vivo* application of γδ T cells. Front Immunol. (2023) 14:1185564. doi: 10.3389/fimmu.2023.1185564. PMID: 37539052 PMC10394837

[30] Hoeres T Smetak M Pretscher D Wilhelm M . Improving the efficiency of Vγ9Vδ2 T-cell immunotherapy in cancer. Front Immunol. (2018) 9:800. doi: 10.3389/fimmu.2018.00800. PMID: 29725332 PMC5916964

[31] Lamb LS Pillai S Langford S Bowersock J Stasi AD Saad A . Clinical-scale manufacturing of γδ T cells for protection against infection and disease recurrence following haploidentical peripheral blood stem cell transplantation and cyclophosphamide gvhd prophylaxis. Bone Marrow Transplant. (2018) 53:766–9. doi: 10.1038/s41409-018-0130-8. PMID: 29515253

[32] Olesti E Bachiller M Oliver-Caldés A ARI program, Hospital Clinic Barcelona . Establishing a sustainable academic point-of-care network for CAR T-cell therapy. Lancet Haematol. (2025) 12:e774–6. doi: 10.1016/S2352-3026(25)00258-3. PMID: 41062203

[33] Burnham RE Zoine JT Story JY Garimalla SN Gibson G Rae A . Characterization of Donor Variability for γδ T Cell ex vivo Expansion and Development of an Allogeneic γδ T Cell Immunotherapy. Front Med. (2020) 7:588453. doi: 10.3389/fmed.2020.588453. PMID: 33282892 PMC7691424

[34] Liang G Hu P Tran N Mahmud H Dash P Schneider D . 253 Fully human CD123 CAR T cells irradicate AML in pre-clinical models and exhibit a favorable safety profile. J Immunother Cancer. (2022) 10. doi: 10.1136/jitc-2022-SITC2022.0253

[35] Pinot L Saßor A Möker N Zhang C Verhoeyen E Hidalgo JV . Transduction of γδ T cells with Baboon envelope pseudotyped lentiviral vector encoding chimeric antigen receptors for translational and clinical applications. Front Immunol. (2025) 16:1548630. doi: 10.3389/fimmu.2025.1548630. PMID: 40547029 PMC12179110

