# Supplementary figures and images for: A clinical-grade automated platform for the manufacturing of CAR-γδ T cells for immunotherapy
Source: Front Immunol. 2026 May 22;17:1779035. doi: 10.3389/fimmu.2026.1779035 (PMC13236643; doi:10.3389/fimmu.2026.1779035)

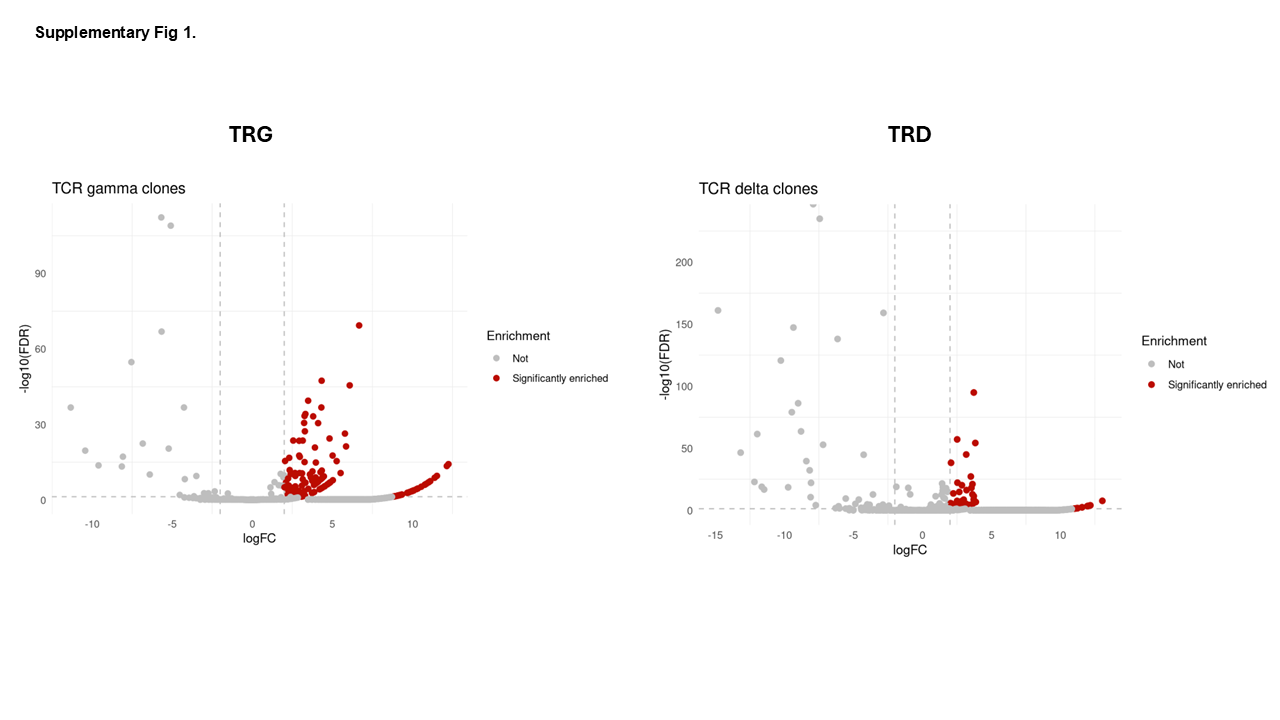

Supplement: Supplementary Figure 1 — The majority of TCR clonotypes maintained stable frequencies throughout cultivation. However, a subset showed significant enrichment by day 14, including 144 TRG and 58 TRD clonotypes. [file Image1.tif]

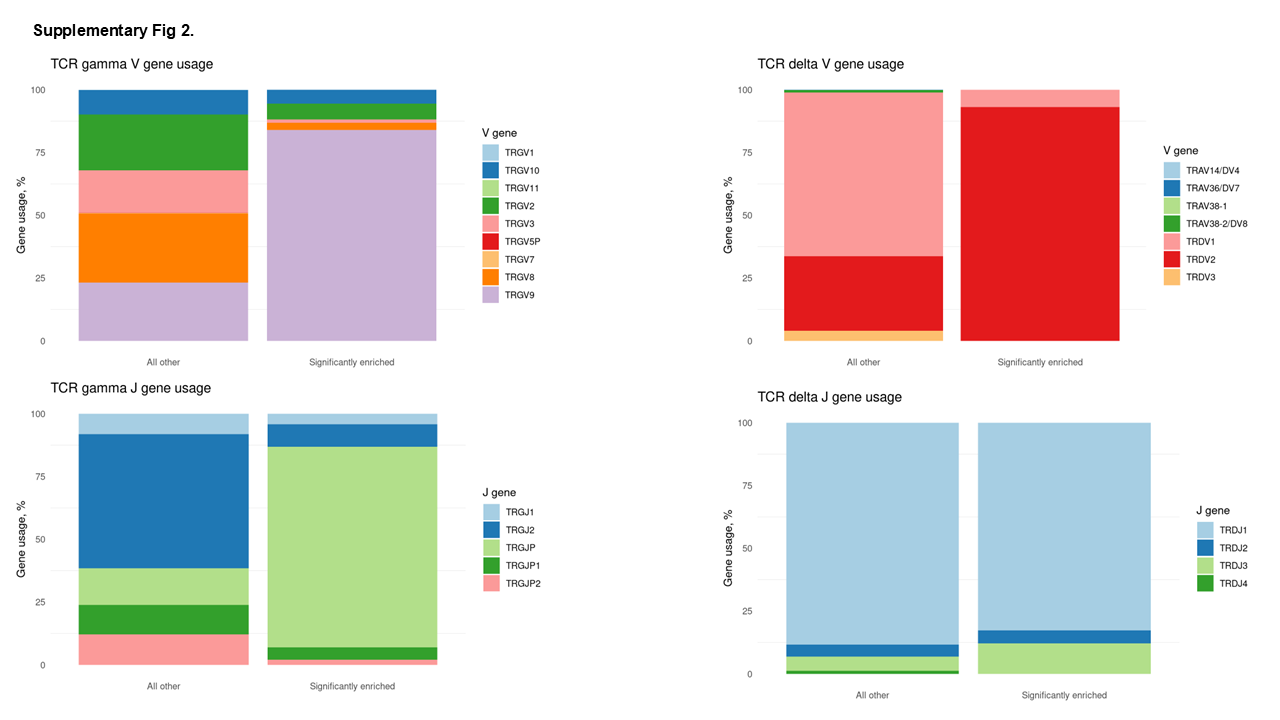

Supplement: Supplementary Figure 2 — Analysis of the enriched TCR gamma clonotypes predominantly utilized TRGV9 and TRGJP gene segments, while the enriched TCR delta clonotypes were mainly characterized by TRDV2 and TRDJ1 usage. [file Image2.tif]

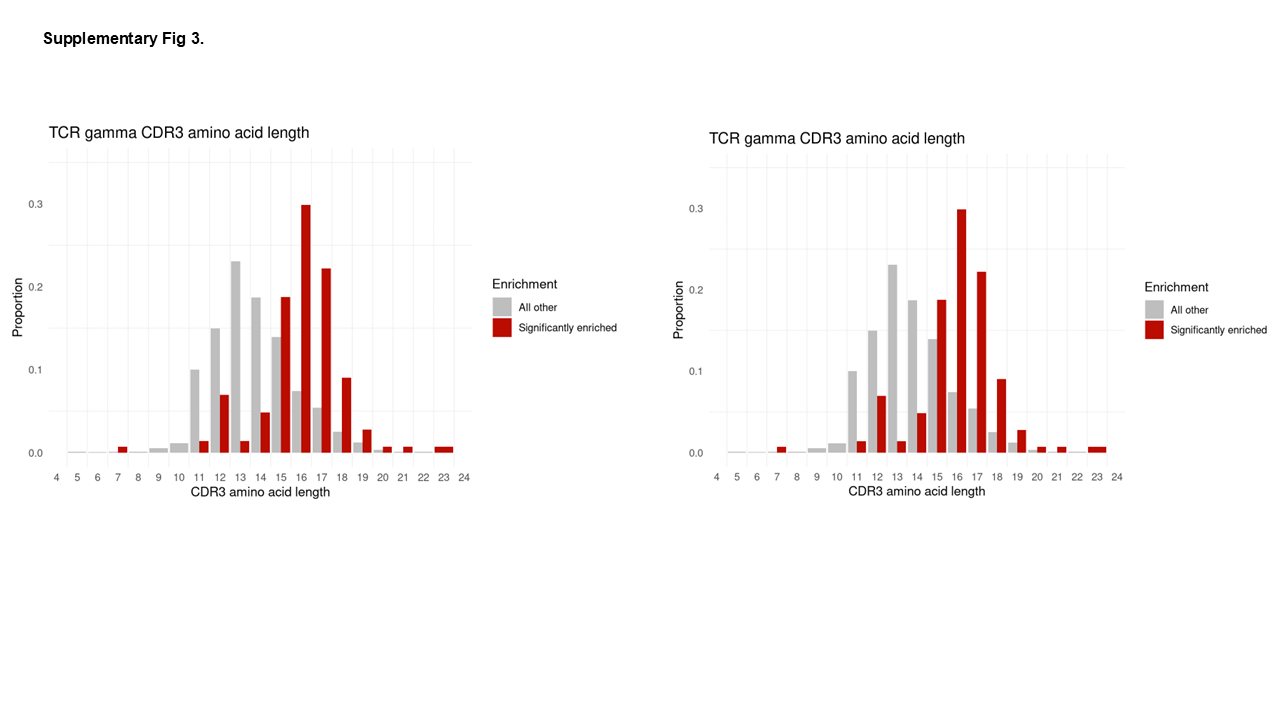

Supplement: Supplementary Figure 3 — Enriched TCR gamma clonotypes exhibited a longer average CDR3 length compared to the complete repertoire, suggesting potential selection for specific structural features during cell culture. [file Image3.tif]

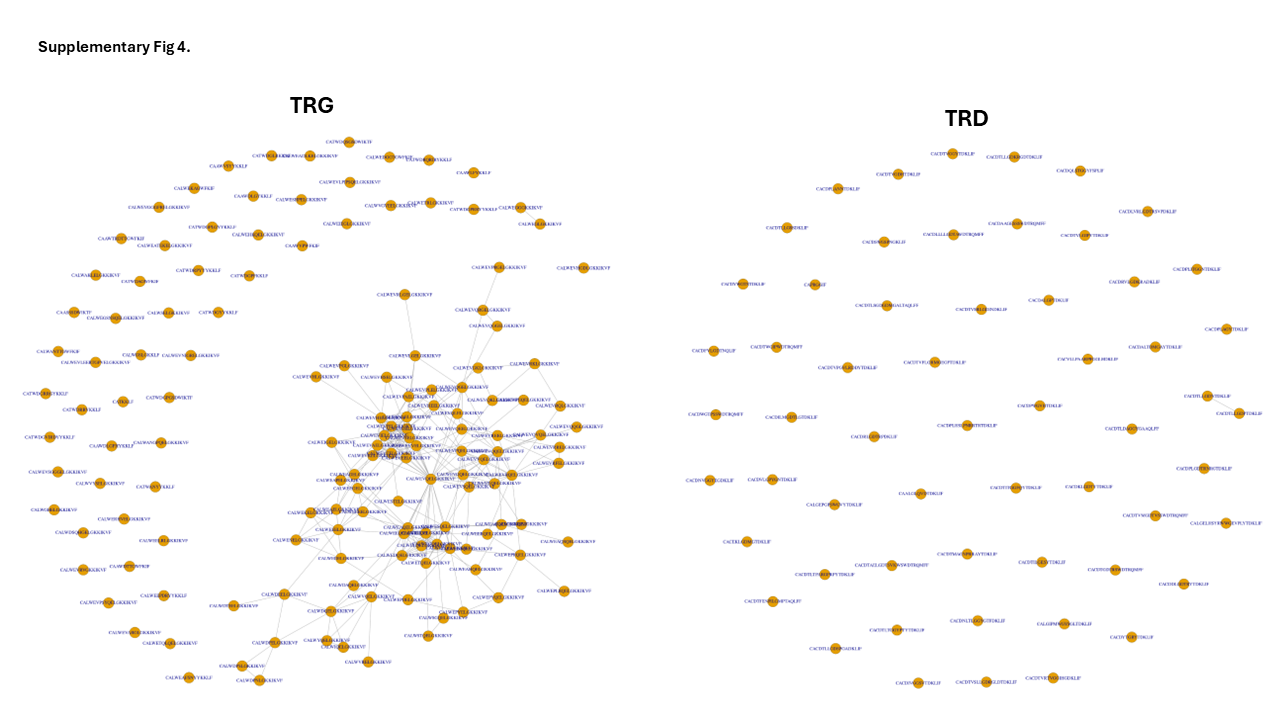

Supplement: Supplementary Figure 4 — A similarity graph based on the CDR3 amino acid sequences of enriched clonotypes was constructed, where clones differing by one or fewer amino acid mismatches were connected. Within the TRG repertoire, a distinct cluster of highly similar clonotypes was observed, indicating potentially shared antigen specificity. [file Image4.tif]

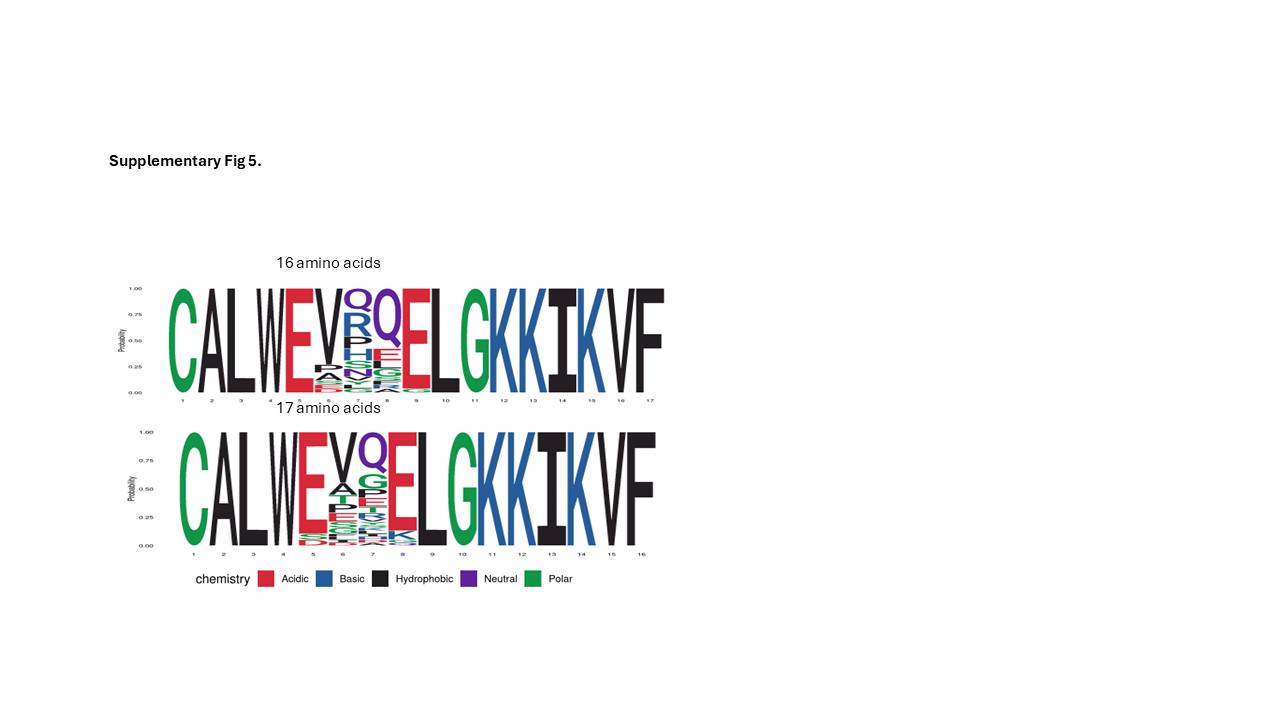

Supplement: Supplementary Figure 5 — The CDR3 amino acid frequency was analyzed for enriched clonotypes that had at least one neighbor in the similarity graph, highlighting conserved sequence features among closely related clones. [file Image5.tif]

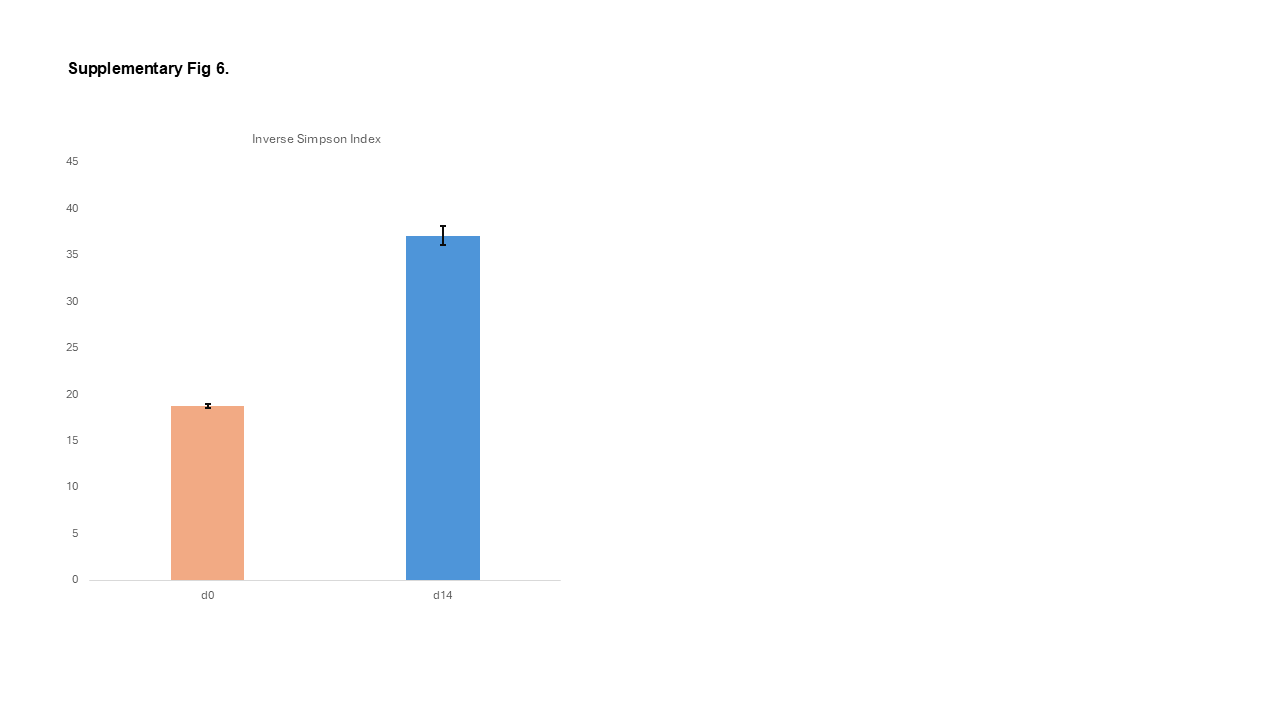

Supplement: Supplementary Figure 6 — The inverse simpson index revealed that the diversity of TCR gamma clonotypes was higher and more evenly distributed on day 14 of cultivation compared to day 0, indicating balanced clonal expansion over time. [file Image6.tif]

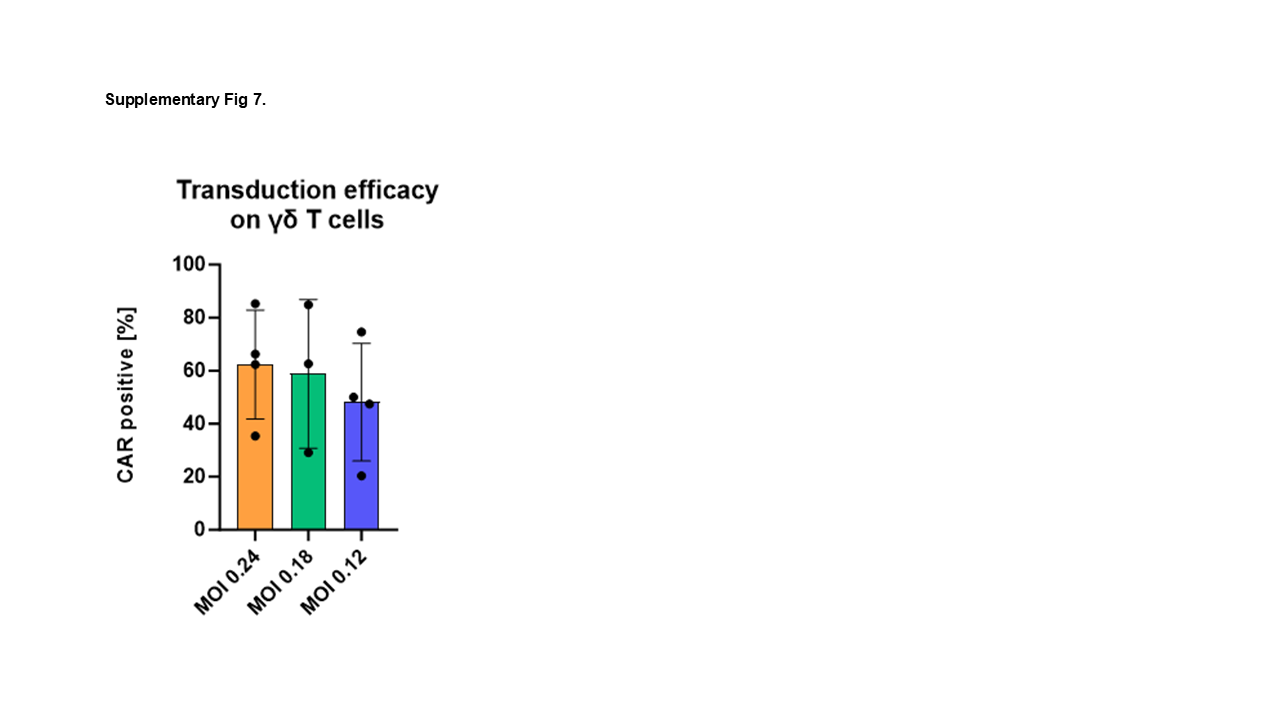

Supplement: Supplementary Figure 7 — CAR transduction efficiency on Vγ9Vδ2 T cells at different multiplicity of infection (MOI) was tested to define the process. [file Image7.tif]

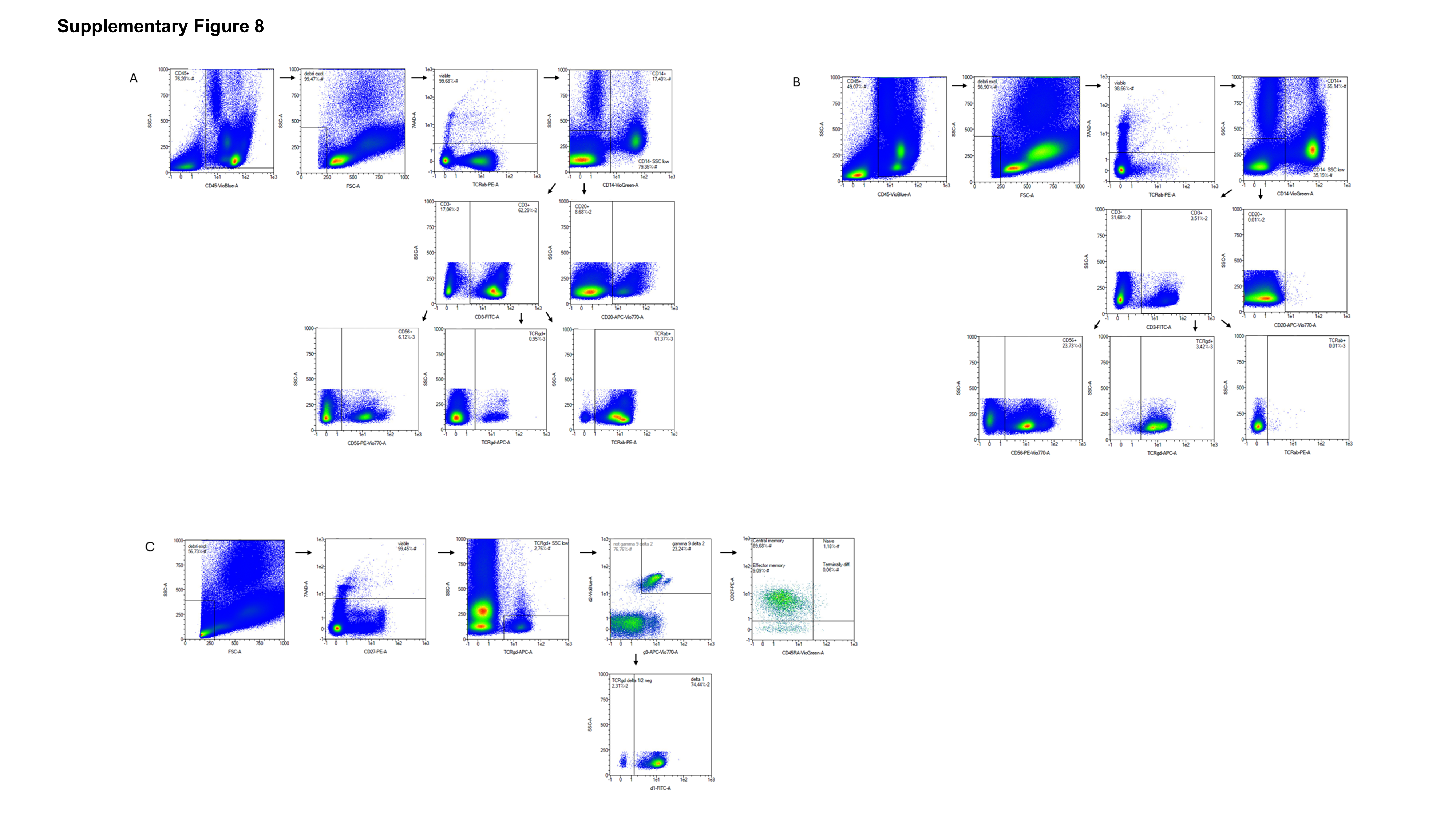

Supplement: Supplementary Figure 8 — Gating strategy used for the flow cytometry analysis of leukapheresis cell composition (A), cell composition after depletion (B), and phenotype of the depleted product (C). [file Image8.tif]
