# Supplementary material for: A clinical-grade automated platform for the manufacturing of CAR-γδ T cells for immunotherapy
Source: Front Immunol. 2026 May 22;17:1779035. doi: 10.3389/fimmu.2026.1779035 (PMC13236643; doi:10.3389/fimmu.2026.1779035)
Supplement: Supplementary file 9 [file Table1.docx]

**Supplementary Tables 1-4.**

**Table 1. List of general reagents**

| **Material** | **Concentration** | **Catalog no.** | **Supplier** |
| --- | --- | --- | --- |
| 7-AAD Staining Solution | 1:10 | 130-111-568 | Miltenyi Biotec |
| anti-CD14-VioGreen (REA599) | 1:50 | 130-110-525 | Miltenyi Biotec |
| Anti-CD16-PEVio 770 (REA423) | 1:50 | 130-113-394 | Miltenyi Biotec |
| Anti-NKG2D-PE (REA797) | 1:50 | 130-111-645 | Miltenyi Biotec |
| anti-Biotin-Vio^®^Bright B515 (REA746) | 1:50 | 130-110-957 | Miltenyi Biotec |
| CD123 CAR detection reagent-Biotin | 1:50 | 130-133-273 | Miltenyi Biotec |
| anti-CD15-PerCP-Vio700 (VIMC6) | 1:50 | 130-113-487 | Miltenyi Biotec |
| anti-CD20-APC-Vio 770 (REA780) | 1:50 | 130-111-341 | Miltenyi Biotec |
| anti-CD27-PE (REA499) | 1:50 | 130-113-640 | Miltenyi Biotec |
| anti-CD3-FITC (REA613) | 1:50 | 130-113-138 | Miltenyi Biotec |
| anti-CD45RA-VioGreen (REA1047) | 1:50 | 130-117-744 | Miltenyi Biotec |
| anti-CD45-VioBlue (REA747) | 1:50 | 130-110-637 | Miltenyi Biotec |
| anti-CD56-PE-Vio 770 (REA196) | 1:50 | 130-113-313 | Miltenyi Biotec |
| anti-CD69-APC-Vio 770 (REA824) | 1:50 | 130-112-616 | Miltenyi Biotec |
| anti-CD8-VioGreen (REA734) | 1:50 | 130-110-684 | Miltenyi Biotec |
| anti-HLA-DR-FITC (REA805) | 1:50 | 130-111-788 | Miltenyi Biotec |
| anti-KIR2DL1-VioGreen (REA1042) | 1:50 | 130-117-481 | Miltenyi Biotec |
| anti-PD1-PE (REA1165) | 1:50 | 130-120-382 | Miltenyi Biotec |
| anti-TCR Vγ9-APC-Vio 770 (REA470) | 1:50 | 130-128-218 | Miltenyi Biotec |
| anti-TCR Vδ1-FITC (REA173) | 1:50 | 130-118-362 | Miltenyi Biotec |
| anti-TCR Vδ2-VioBlue (REA771) | 1:50 | 130-111-015 | Miltenyi Biotec |
| anti-TCRα/β-PE (BW242/412) | 1:50 | 130-133-983 | Miltenyi Biotec |
| anti-TCRγ/δ-APC (REA591) | 1:50 | 130-113-508 | Miltenyi Biotec |
| anti-TIGIT-PE-Vio 615 (REA1004) | 1:50 | 130-116-816 | Miltenyi Biotec |
| anti-TIM3-FITC (REA635) | 1:50 | 130-124-442 | Miltenyi Biotec |
| BM-Lactate test strips | n.a | 03012654016 | Roche |
| CliniMACS Prodigy® Tubing Set 320 | n.a | 200-073-615 | Miltenyi Biotec |
| CliniMACS Prodigy® Tubing Set 620 | n.a | 200-073-620 | Miltenyi Biotec |
| CliniMACS® CD19 Reagent | n.a. | 200-070-117 | Miltenyi Biotec |
| CliniMACS® PBS/EDTA Buffer CR/GMP | n.a. | 200-070-022 | Miltenyi Biotec |
| CliniMACS® TCRα/β-Biotin CR/GMP | n.a. | 220-001-946 | Miltenyi Biotec |
| CliniMACS® Anti-Biotin Reagent | n.a. | 200-070-120 | Miltenyi Biotec |
| DNeasy Blood & Tissue Kit | n.a | 69506 | Qiagen |
| Human Male AB Serum | 5% | n.a. | Grifols Bio Supplies Inc |
| Lentiviral vector aCD123 CAR | MOI 0.12 | n.a. | Miltenyi Biotec |
| MACS® COPYcheck Kit | n.a | 130-128-157 | Miltenyi Biotec |
| MACS® GMP Recombinant Human IL-15 | 140 IU/ml | 170-076-114 | Miltenyi Biotec |
| MACS® GMP Recombinant Human IL-2 | 500 IU/ml | 170-076-147 | Miltenyi Biotec |
| MACS® GMP Vectofusin®-1 | 10 µg/ml | 170-076-165 | Miltenyi Biotec |
| MiLab Human g & d TCR RNA Multiplex Kit | n.a. | n.a. | MiLaboratories |
| NEB Next Library Quant Kit | n.a. | E7630S | New England Biolabs |
| Propidium Iodide Solution | 1 μg/mL | 130-093-233 | Miltenyi Biotec |
| Qubit dsDNA HS Assay kit | n.a. | Q32851 | Thermo Fischer Scientific |
| Qubit RNA HS Assay kit | n.a. | Q32852 | Thermo Fischer Scientific |
| RNeasy Mini Kit | n.a. | 74104 | Qiagen |
| TexMACS™ GMP Medium | n.a. | 170-076-306 | Miltenyi Biotec |
| Zometa® | 2 µg/ml | PZN 08830333 | Novartis |

**Table 2. List of equipment.**

| **Equipment** | **Supplier** | **Additional information** |
| --- | --- | --- |
| Accutrend Plus system | Roche | n.a. |
| CliniMACS Prodigy® | Miltenyi Biotec | n.a. |
| GlucCell glucose meter | Cesco Bioengineering | n.a. |
| Illumina MiSeq | Illumina | n.a. |
| Illumina NextSeq 2000 | Illumina | n.a. |
| MACSQuant^®^ Analyzer 10 flow cytometer | Miltenyi Biotec | MACSQuantify Software version 2.13 |
| RNA 6000 Nano Chip | Agilent | n.a. |
| Seven2Go pH meter | Mettler-Toledo | n.a. |

**Table 3. Flow cytometric panel design for the MACSQuant flow analyzer 10.**

| **Panel** | **Reagent** | **Concentration** | **Supplier** |
| --- | --- | --- | --- |
| 1 | 7-AAD Staining Solution | 1:10 | Miltenyi Biotec |
|  | anti-CD45-VioBlue (REA747) | 1:50 | Miltenyi Biotec |
|  | anti-TCRγ/δ-APC (REA591) | 1:50 | Miltenyi Biotec |
| 2 | 7-AAD Staining Solution | 1:10 | Miltenyi Biotec |
|  | anti-CD45-VioBlue (REA747) | 1:50 | Miltenyi Biotec |
|  | anti-CD14-VioGreen (REA599) | 1:50 | Miltenyi Biotec |
|  | anti-CD3-FITC (REA613) | 1:50 | Miltenyi Biotec |
|  | anti-TCRα/β-PE (BW242/412) | 1:50 | Miltenyi Biotec |
|  | anti-CD56-PE-Vio 770 (REA196) | 1:50 | Miltenyi Biotec |
|  | anti-TCRγ/δ-APC (REA591) | 1:50 | Miltenyi Biotec |
|  | anti-CD20-APC-Vio 770 (REA780) | 1:50 | Miltenyi Biotec |
| 3.1 | CD123 CAR detection reagent-Biotin | 1:50 | Miltenyi Biotec |
| 3.2 | 7-AAD Staining Solution | 1:10 | Miltenyi Biotec |
|  | anti-CD45-VioBlue (REA747) | 1:50 | Miltenyi Biotec |
|  | anti-CD14-VioGreen (REA599) | 1:50 | Miltenyi Biotec |
|  | anti-Biotin-Vio^®^Bright B515 (REA746) | 1:50 | Miltenyi Biotec |
|  | anti-TCRα/β-PE (BW242/412) | 1:50 | Miltenyi Biotec |
|  | anti-CD56-PE-Vio 770 (REA196) | 1:50 | Miltenyi Biotec |
|  | anti-TCRγ/δ-APC (REA591) | 1:50 | Miltenyi Biotec |
|  | anti-CD20-APC-Vio 770 (REA780) | 1:50 | Miltenyi Biotec |
| 4 | 7-AAD Staining Solution | 1:10 | Miltenyi Biotec |
|  | anti-TCR Vγ9-APC-Vio 770 (REA470) | 1:50 | Miltenyi Biotec |
|  | anti-TCR Vδ1-FITC (REA173) | 1:50 | Miltenyi Biotec |
|  | anti-TCR Vδ2-VioBlue (REA771) | 1:50 | Miltenyi Biotec |
|  | anti-CD45RA-VioGreen (REA1047) | 1:50 | Miltenyi Biotec |
|  | anti-CD27-PE (REA499) | 1:50 | Miltenyi Biotec |
|  | anti-TCRγ/δ-APC (REA591) | 1:50 | Miltenyi Biotec |
| 5 | 7-AAD Staining Solution | 1:10 | Miltenyi Biotec |
|  | anti-TCR Vδ2-VioBlue (REA771) | 1:50 | Miltenyi Biotec |
|  | anti-CD8-VioGreen (REA734) | 1:50 | Miltenyi Biotec |
|  | anti-HLA-DR-FITC (REA805) | 1:50 | Miltenyi Biotec |
|  | Anti-NKG2D-PE (REA797) | 1:50 | Miltenyi Biotec |
|  | Anti-CD16-PEVio 770 (REA423) | 1:50 | Miltenyi Biotec |
|  | anti-TCRγ/δ-APC (REA591) | 1:50 | Miltenyi Biotec |
|  | anti-CD69-APC-Vio 770 (REA824) | 1:50 | Miltenyi Biotec |
| 6 | 7-AAD Staining Solution | 1:10 | Miltenyi Biotec |
|  | anti-TCR Vδ2-VioBlue (REA771) | 1:50 | Miltenyi Biotec |
|  | anti-KIR2DL1-VioGreen (REA1042) | 1:50 | Miltenyi Biotec |
|  | anti-TCRγ/δ-APC (REA591) | 1:50 | Miltenyi Biotec |
|  | anti-TIGIT-PE-Vio 615 (REA1004) | 1:50 | Miltenyi Biotec |
|  | anti-TIM3-FITC (REA635) | 1:50 | Miltenyi Biotec |
|  | anti-PD1-PE (REA1165) | 1:50 | Miltenyi Biotec |

**Table 4. CliniMACS Prodigy activity matrix for the stimulation and expansion of γδ T cells.**
